# A simple and efficient dual optical signaling chemodosimeter for toxic Hg(II)

**DOI:** 10.3762/bjoc.8.155

**Published:** 2012-08-22

**Authors:** Sabir H Mashraqui, Sapna A Tripathi, Sushil S Ghorpade, Smita R Britto

**Affiliations:** 1 Department of Chemistry, University of Mumbai, Vidyanagari, Mumbai-400098, India

**Keywords:** chemidosimeter, chemosensor, fluorescence, Hg(II) selective, UV–visible, visual detection

## Abstract

10-Methylthioacridone, designated as acrithion, has been employed as an easily accessible chemodosimeter for the optical targeting of toxic Hg^2+^ in buffered aqueous DMSO. The Hg^2+^-mediated desulfurization of the probe is translated into selective dual signaling of Hg^2+^ by means of color bleaching and fluorescence amplification while several other metal ions, including potentially competing Ag^+^, Cu^2+^ and Pb^2+^, afford no significant interferences even in excess concentrations.

## Introduction

Interest continues unabatedly to design optical probes for the selective detection of metal ions of importance across disciplines of biology, medicine and environment [1–5]. Though, mercury and alkylmercury are highly poisonous, mercury ions are also a subject of worldwide concern because of their acute immune-, geno- and neurotoxic effects on human, livestock and marine mammals [6–11]. The poisonous effects of mercury ions stem from their irreversible binding with the sulfhydryl-containing groups of proteins and enzymes, resulting in clinical problems such as prenatal brain damage, muscle coordination difficulties, lung, kidney and vision disorders.

To date, many chemosensors featuring assortments of ligands and signalling units have been reported for the detection of potentially toxic metal ions such as Cu^2+^, Hg^2+^, Pb^2+^ etc. However, owing to the reversible coordination with the probes, these paramagnetic metal ions often result in less desirable fluorescence quenching effects [12–16]. Lately, the chemodosimeter-based sensing has gained increasing popularity on account of the irreversible chemical intervention of analytes with the probe molecules. Since the metal ions are disengaged from the chemically modified dosimeters, the process results in the more reliable fluorescence ratiometric or switch-on responses even for the paramagnetic metal ions [17–19]. The first luminescence chemodosimeter for Hg^2+^ was developed by Czarnik et al. and was based on the Hg^2+^-mediated desulfurization of anthracene-thioamide chromophore [20–21]. Following this pioneering report, several other groups have utilized the exceptionally strong thiophilicity of Hg^2+^ to access a range of luminescent Hg^2+^ chemodosimeters [22–38]. Despite impressive advances [39–40], many known Hg^2+^ chemosensors suffer from multistep syntheses and delayed responses or cross affinities, especially from Ag^+^, Pb^2+^ and Cu^2+^ [41–51]. Consequently, designing easily accessible Hg^2+^-selective chemosensors with dual colorimetric and fluorescence switch-on capabilities is deemed of interest in supramolecular research.

Acridone, a photostable fluorophore [52], has been designed to function as metal ion and anion sensor [53–57]. In contrast to moderately fluorescent acridone, its thione analog is poorly emissive presumably on account of the intersystem crossing process of the C=S bonding [58]. Keeping this in mind, we have presently conceived the design of simple and easily accessible 10-methylthioacridone – designated as acrithion **2** – as a new chemodosimeter for the highly selective colorimetric and fluorescence signaling of Hg^2+^.

**Scheme 1:**
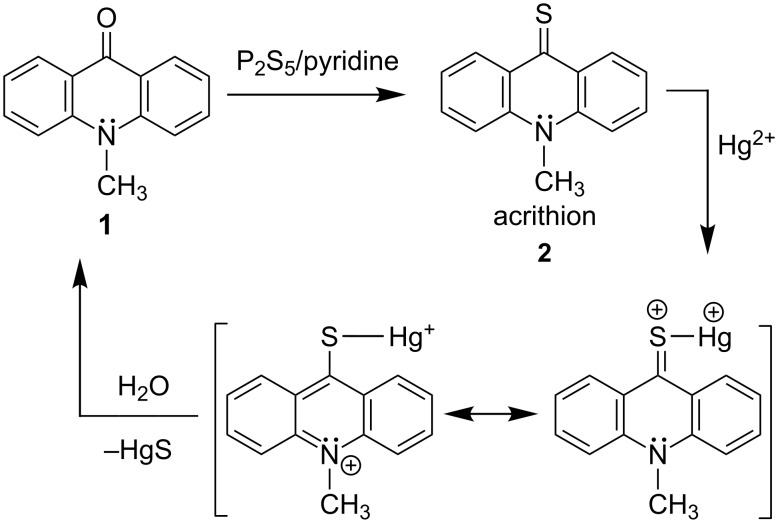
Synthesis of acrithion **2** and its chemodosimeter mechanism for Hg^2+^ recognition.

The chemodosimeter mechanism, illustrated in Scheme 1, is proposed to proceed via chelation of Hg^2+^ with the thione group of acrithion **2**, forming an intermediate thioacridinium–Hg^+^ complex. This phenomena would polarize the C=S bond and therefore facilitate nucleophilic attack by water at the electrophilic C9 position. Subsequently, the hydrolytic desulfurization is expected to form compound **1** – which is more fluorescent – at the expense of poorly emitting acrithion **2**. In addition to the fluorescence amplification, the difference in the absorbance wavelengths between acrithion **2** and the desulfurization product **1** would also afford colorimetric naked-eye signaling of Hg^2+^.

## Results and Discussion

**Figure 1:**
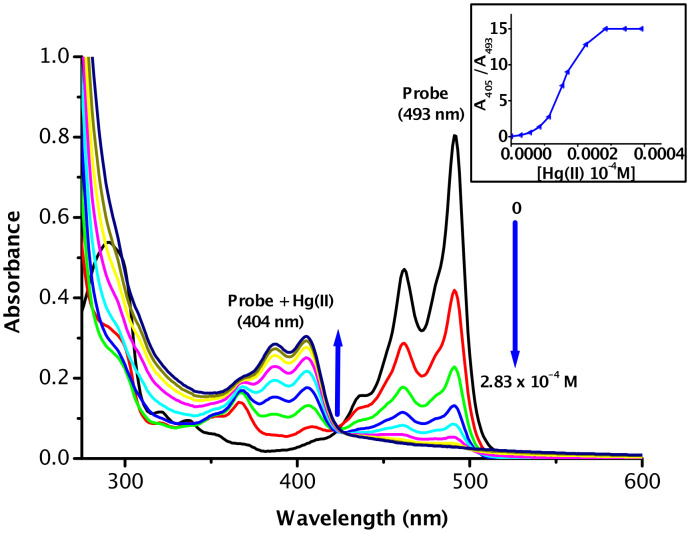
Spectrophotometric titration of acrithion **2** (2.83 × 10^−5^ M) with Hg^2+^ (0–2.83 × 10^−4^ M) in DMSO–H_2_O (70:30 v/v) at pH 7.4.

Acrithion **2** is synthesized in good yield by treatment of readily available 10-methyl acridone **1** with P_2_S_5_ in pyridine [59] (Supporting Information File 1). The optical sensitivities of acrithion **2** towards various biologically and environmentally important metal ions as their perchlorates were evaluated on the basis of UV–vis as well as fluorescence spectral responses. Acrithion **2** (2.83 × 10^−5^ M) displayed absorption bands at 493, 462 and 293 nm in DMSO–H_2_O (70:30 v/v) at pH 7.4 (10 mM HEPES buffer). As shown in Figure 1, the spectrophotometric titration of acrithion **2** (2.83 × 10^−5^ M) with increasing Hg^2+^ induced a new blue-shifted maximum at 404 nm, while the probe’s maxima at 493 and 462 nm gradually diminished and disappeared at the limiting 2.83 × 10^−4^ M of Hg^2+^. At the limiting concentration of Hg^2+^, the final UV–vis spectrum was essentially similar to that of **1** [60], an observation that supports the Hg^2+^-induced desulfurization of the probe [60]. An independent confirmation of **1** was also obtained from the ^13^C NMR spectrum of the product isolated from the reaction of acrithion **2** with Hg(ClO_4_)_2_ in aqueous DMSO (Supporting Information File 1).

**Figure 2:**
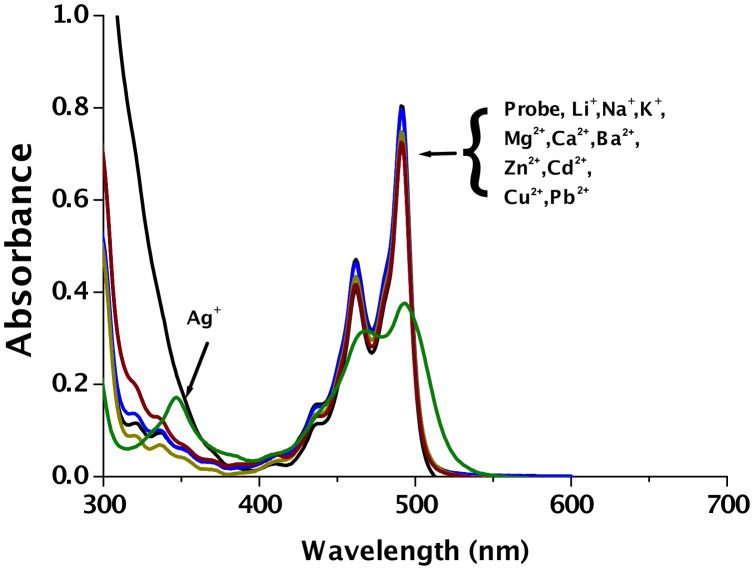
Spectrophotometric investigation of acrithion **2** (2.83 × 10^−5^ M) with metal ions (2.83 × 10^−3^ M) in DMSO–H_2_O (70:30 v/v) at pH 7.4.

As depicted in Figure 2, no detectable changes in the absorbance profile of acrithion **2** could be seen up on adding 2.83 × 10^−3^ M of Li^+^, Na^+^, K^+^, Mg^2+^, Ca^2+^, Ba^2+^, Co^2+^, Ni^2+^, Cu^2+^, Zn^2+^, Cd^2+^, and Pb^2+^ even after 20 min, implying the absence of substantive ground state interaction. On the other hand, the exposure of the probe to Ag^+^ at 2.83 × 10^−3^ M led to partial reductions of the probe’s maxima at 493 and 462 nm, accompanied by the appearance of a new, weakly absorbing band at 347 nm. However, unlike Hg^2+^, the desulfurization product **1** could not be detected with Ag^+^. Though it has not been investigated in detail, the findings mentioned above imply that the interaction of Ag^+^ is not only relatively weaker, but also of a different nature than observed with Hg^2+^. The addition of Hg^2+^ turned the deep yellow solution of acrithion **2** into a colorless one, permitting ready naked-eye detection of this ion. While weakly interacting Ag^+^ induced only a slight dilution of the yellow color, no perceptible color modulations were noticeable with the several background metal ions which were examined (Supporting Information File 1).

**Figure 3:**
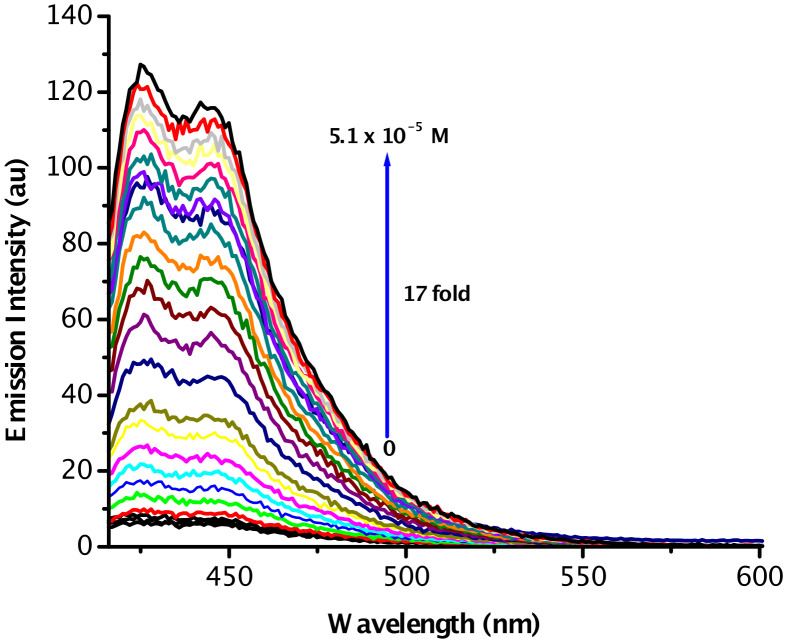
Fluorimetric titration of acrithion **2** (2.5 × 10^−6^ M) with Hg^2+^ (0–5.1 × 10^−5^ M) in DMSO–H_2_O (70:30 v/v) at pH 7.4.

Excitation of acrithion **2** at 404 nm in DMSO–H_2_O (70:30 v/v) at pH 7.4 gave rise to a weak emission band at 424 nm with a quantum yield (Ф_F_) of 0.021, calculated with respect to anthracene (Ф_F_ = 0.27) [61]. Fluorimetric titration (Figure 3) revealed linear enhancements in the emission intensity with a significant 17-fold enhancement observed at a limiting 5.1 × 10^−5^ M of Hg^2+^ [60]. Based on the linear response observed in emission intensity with respect to increasing Hg^2+^ concentration, we calculated the limit of detection (LOD) of Hg^2+^ to be 1.06 × 10^−8^ mol L^−1^ or 2.12 μg L^−1^ of Hg^2+^ (Supporting Information File 1). Though, this LOD level of the probe is unsuitable to measure 1 μg L^−1^ of Hg^2+^ – the lowest permission quantity in drinking water – however, there might be potential for measuring micromolar concentrations of Hg^2+^ in environmental samples with the probe.

In contrast to the sizeable fluorescence amplification observed with Hg^2+^, the weakly interacting Ag^+^ induced only less marked emission enhancement of only 3-fold at a concentration which was nearly 10 times the limiting concentration of Hg^2+^ (Supporting Information File 1). Consistent with their lack of ground state interaction, metal ions viz. Li^+^, Na^+^, K^+^, Mg^2+^, Ca^2+^, Ba^2+^, Co^2+^, Ni^2+^, Cu^2+^, Zn^2+^, Cd^2+^, and Pb^2+^ up to 100 equiv did not reveal any significant fluorescence modulations (Supporting Information File 1).

**Figure 4:**
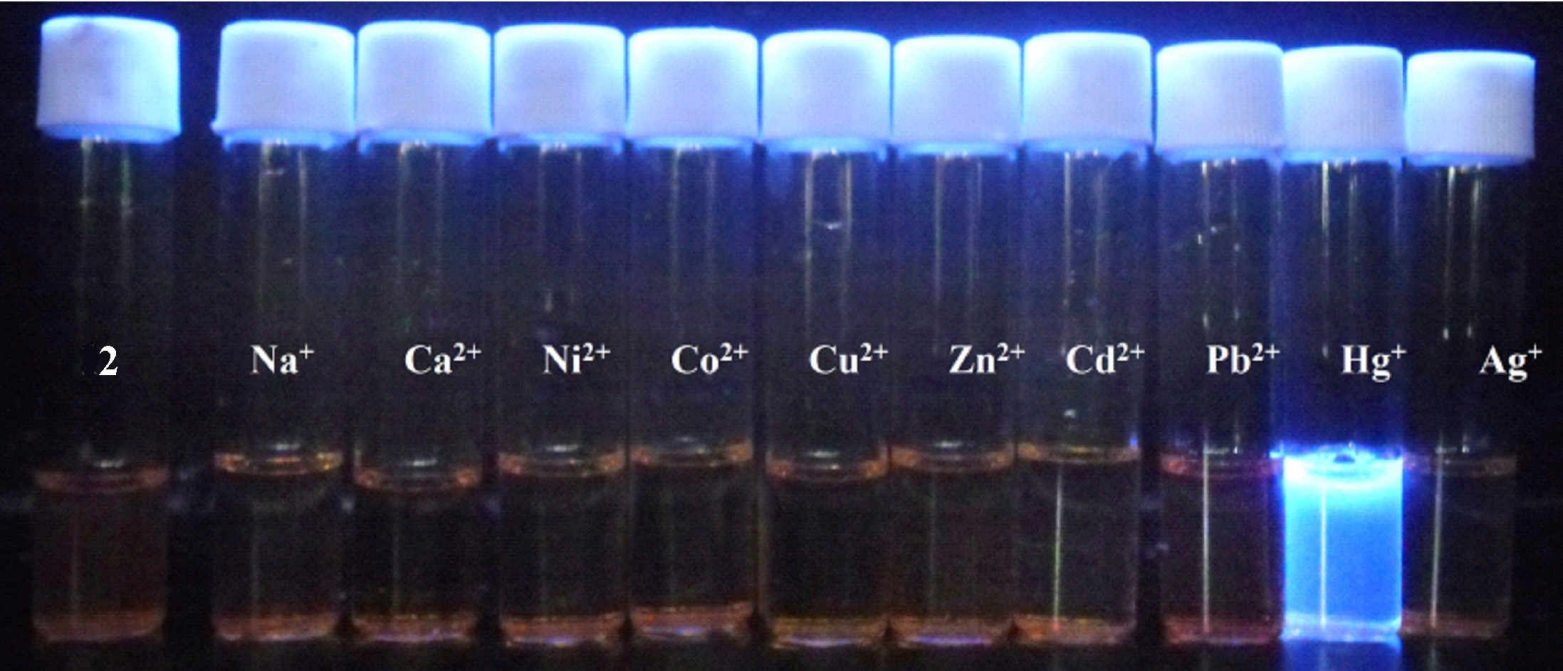
Changes in fluorescence of acrithion **2** upon addition of metal ions in DMSO–H_2_O (70:30 v/v) at pH 7.4.

**Figure 5:**
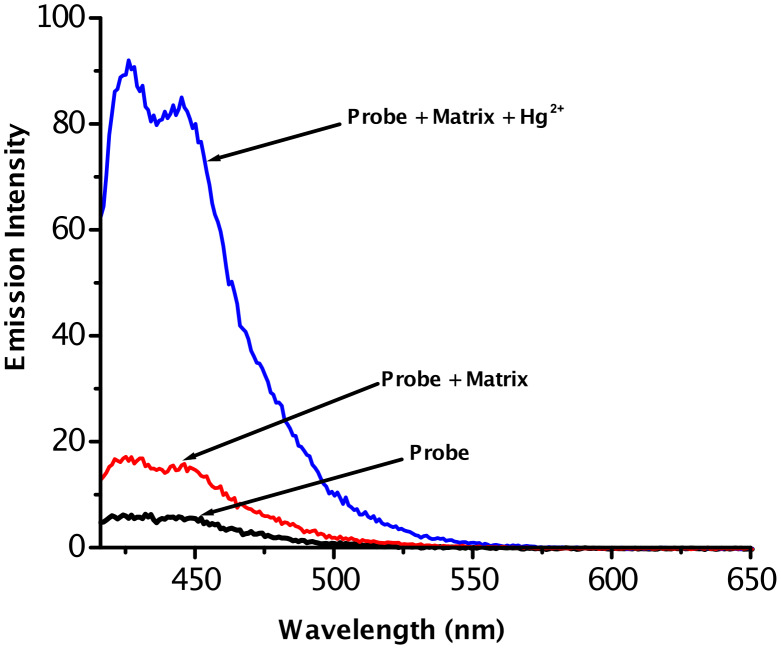
Emission spectra of acrithion **2** (2.5 ×10^−6^ M), acrithion **2** + matrix consisting of 2.5 × 10^−4^ M each of Li^+^, Na^+^, K^+^, Mg^2+^, Ca^2+^, Ba^2+^, Co^2+^, Ni^2+^, Cu^2+^, Zn^2+^, Cd^2+^, Ag^+^ and Pb^2+^ and acrithion **2** + matrix + Hg^+2^ (5.1 × 10^−5^ M).

As shown in Figure 4, the addition of Hg^2+^ to a solution of acrithion **2** generated a brilliant blue fluorescence whereas other metal ions showed no detectable fluorescence responses. To verify the high selectivity of Hg^2+^ over other metal ions, we first measured the fluorescence response of the probe (2.5 × 10^−6^ M) in the presence of a matrix consisting of 100 equiv of each Li^+^, Na^+^, K^+^, Mg^2+^, Ca^2+^, Ba^2+^, Co^2+^, Ni^2+^, Cu^2+^, Zn^2+^, Cd^2+^, Ag^+^ and Pb^2+^. The matrix gave rise to about 3-fold fluorescence enhancement compared to that of the free probe. The addition of Hg^2+^ (5.1 × 10^−5^ M) to the matrix solution described above caused ca. 15-fold fluorescence enhancement, a value comparable to that induced by Hg^2+^ alone at the same concentration. This experiment (Figure 5) clearly demonstrates the superior chemodosimeter action of hazardous mercury ions in samples containing several background metal ions.

**Figure 6:**
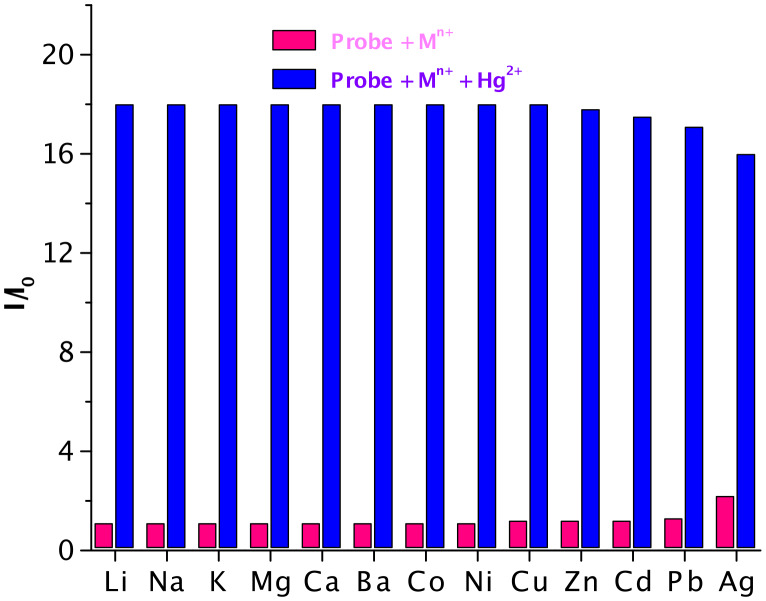
Fluorimetric response of acrithion **2** (2.5 × 10^−6^ M) in the presence of background metal ions (2.5 × 10^−4^ M each) in red and after addition of Hg^2+^ (5.1 × 10^−5^ M) in blue.

In addition to the matrix experiment described above, we studied the effects of individual interferents on the fluorescence response of mercury ions. Therefore, the fluorescence spectra of the probe in the presence of individual metal ions (2.5 × 10^−4^ M each) without and with added merucury ions at 5.1 × 10^−5^ M were recorded. As shown in Figure 6, the background metal ions exhibited either none or insignificant perturbations on the selective fluorescence response observed upon adding mercury ions.

**Figure 7:**
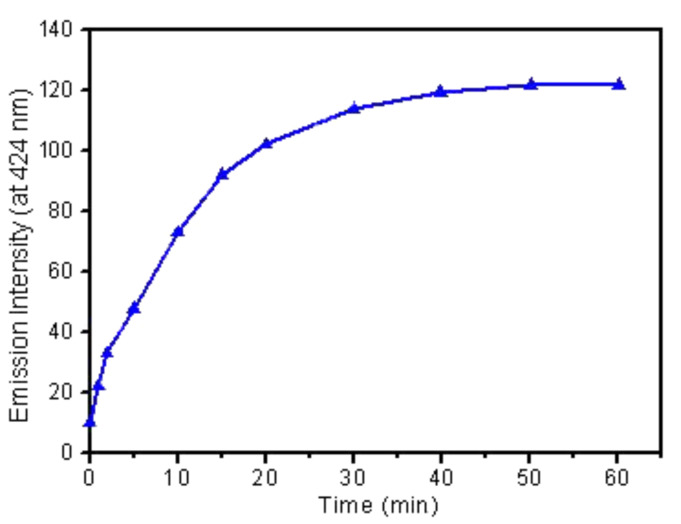
Time course plot of the change in emission intensity of acrithion **2**.

Next, to evaluate the response time, we plotted a time-dependent evolution of fluorescence with respect to the limiting concentration of Hg^2+^ at 20 °C. As shown in Figure 7, a substantial gain in the emission intensity (ca. 75 % of the total enhancement) is recorded under 20 min of contact time. Thereafter, the fluorescence enhancement occurs relatively slowly, reaching an optimum value within ca. 30 min and then remaining steady beyond this point. The response time of acrithion **2** is comparable to many known Hg^2+^ chemodosimeters, however, this delayed time for Hg^2+^ signaling may limit its practical applications.

## Conclusion

In conclusion, 10-methylthioacridone constitutes one of the simplest and most easily accessible chemodosimeters affording dual colorimetric and fluorescence switch-on responses for the selective and sensitive targeting of toxic Hg^2+^. Other merits of the chemodosimeter include visible-light excitation and absence of significant interferences, especially from the competing Ag^+^, Cu^2+^ and Pb^2+^, the features which may prove conducive to Hg^2+^-sensing applications in certain circumstances.

## Supporting Information

File 1 Synthesis of acrithion **2**, IR, ^1^H NMR, and ^13^C NMR data, experimental confirmation of 10-methylacridone, naked-eye detection and the determination of the limit of detection.
