# Supplementary material for: A simple and efficient dual optical signaling chemodosimeter for toxic Hg(II)
Source: Beilstein J Org Chem. 2012 Aug 22;8:1352–7. doi: 10.3762/bjoc.8.155 (PMC3458760; doi:10.3762/bjoc.8.155)
Supplement: File 1 — Synthesis of acrithion 2, IR, 1H NMR, and 13C NMR data, experimental confirmation of 10-methylacridone, naked-eye detection and the determination of the limit of detection. [file Beilstein_J_Org_Chem-08-1352-s001.pdf]

Supporting Information

for

A simple and efficient dual optical signaling chemodosimeter for toxic Hg(II)

Sabir H. Mashraqui*, Sapna A. Tripathi, Sushil S. Ghorpade and Smita R. Britto

Address: Department of Chemistry, University of Mumbai, Vidyanagari, Mumbai-400098, India

Email: Sabir H. Mashraqui. sh_mashraqui@yahoo.com

*Corresponding author

Synthesis of acrithion 2, IR, ^{1}H NMR, and ^{13}C NMR data, experimental confirmation of 10-methylacridone, naked-eye detection and the determination of the limit of detection.

Contents

1) Materials and synthesis of acrithion **2** S2
2) IR spectrum of acrithion **2** S3
3) ^{1}H NMR of acrithion **2** S3
4) ^{13}C NMR spectrum of acrithion **2** S4
5) ^{13}C NMR of acrithion **2** without and with Hg^{2+} S4
6) Experimental confirmation of the formation of 10-methylacridone (**1**) S5
7) Visual detection S5
8) Detection limit S5
9) References S6

Materials and synthesis:

Chemicals and solvents used were purchased from S. D. Fine Chemicals or Sigma-Aldrich, India. UV–vis spectra were recorded using a Shimadzu UV–vis. spectrophotometer, model no. UV-2450. Fluorescence studies were carried out using a Shimadzu spectrofluorophotometer RF-5301PC. IR was recorded using Perkin Elmer FT-IR spectrometer and pellets were made by using KBr. ^{1}H NMR were recorded on a Bruker Avance II 300 at 300 MHz.

Synthesis of acrithion 2: 10-Methylacridone (**1**, 209 mg, 1 mmol) was heated with P_2S_5 (222 mg, 1 mmol) in 5 mL dry pyridine at 80 °C for 8 h. After completion, the reaction mixture was diluted with water. The precipitated solid was filtered, washed thoroughly with water and dried under vacuum. Repeated crystallization from dry methanol afforded acrithion **2** as orange colored solid (158 mg; 70%); mp 222–225 °C (lit. 224 °C) [1]; IR (KBr): 2923, 1601 1574, 1222, 989, 744 cm^{-1} (Figure S1); ^{1}H NMR (DMSO-d_6, 300 MHz) δ 8.9 (dt, *J* = 8.1 Hz, 0.6 Hz, 2H), 8.0 (dd, *J* = 8.7 Hz, 1.5 Hz, 2H), 7. 92–7. 87 (td, *J* = 6.6 Hz, 1.5 Hz, 2H), 7.45–7.40 (td, *J* = 8.4 Hz, 0.9Hz, 2H), 4.08 (s, 3H, (Figure S2); ^{13}C NMR (DMSO-d_6) δ 198.57, 138.0, 135.12, 130.93, 130.54, 123.70, 117.54, 35.44 ppm. (Figure S3); Anal. cald. for $C_{14}H_{11}NS$: C, 74.66; H 4.88; N, 6.22; S, 14.22; found:- C, 74.32; H, 4.54; N, 6.08; S, 14.03% .

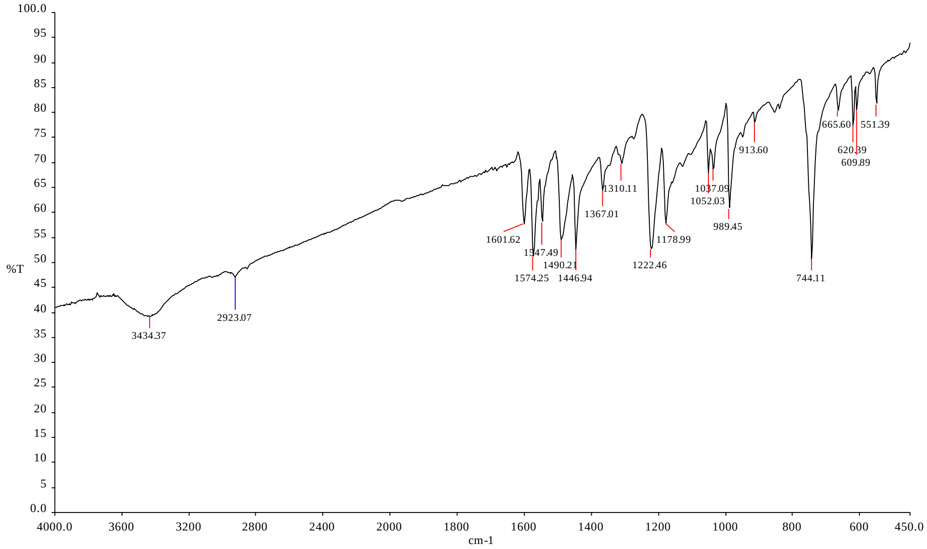

Figure S1: IR spectrum of acrithion **2**.

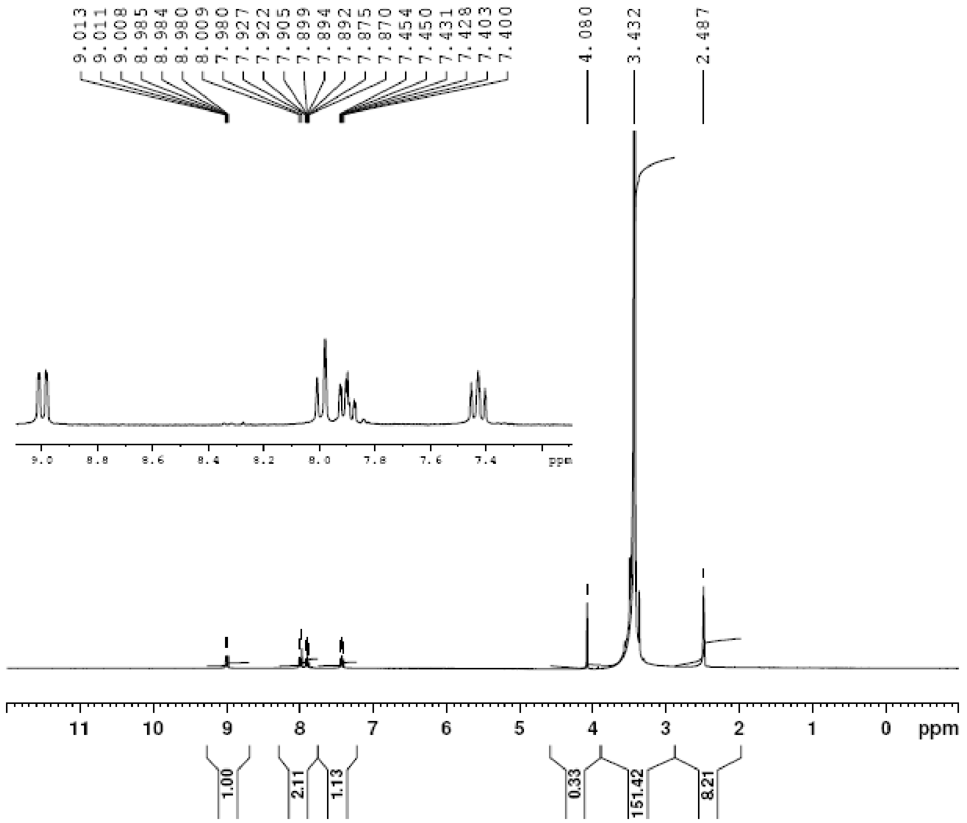

Figure S2: ^{1}H NMR spectrum of acrithion **2** (DMSO-d_6).

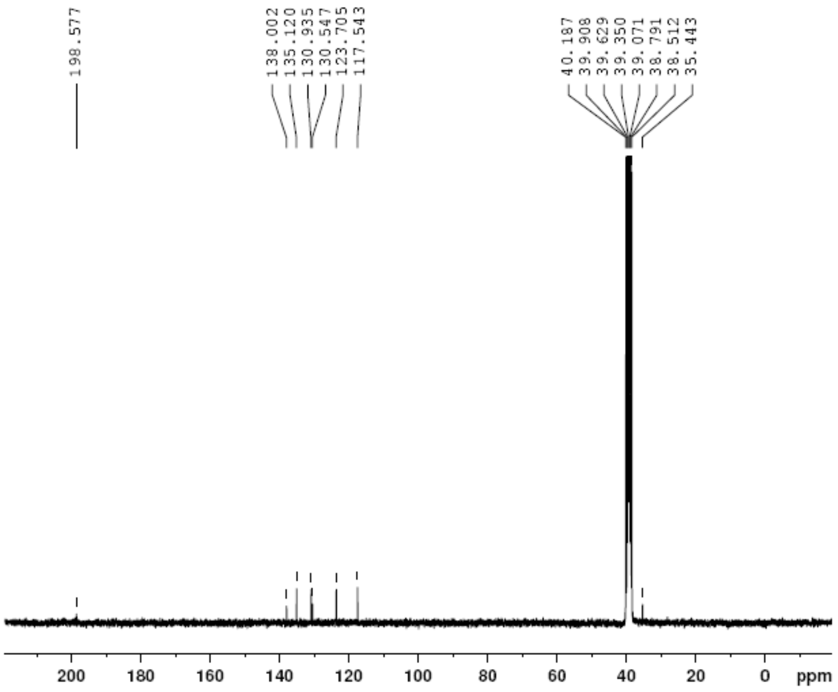

Figure S3: ^{13}C NMR spectrum of acrithion **2** (DMSO-d_6)

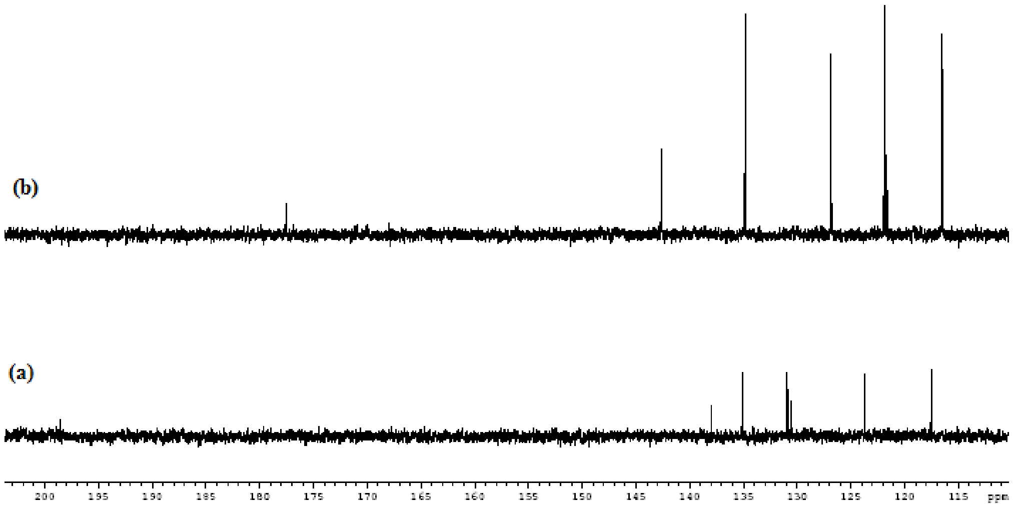

Figure S4: ^{13}C NMR, (a) acrithion **2** only (b) acrithion **2** + $Hg(ClO_4)_2$ (5 equiv) in DMSO(d_6)–D_2O (9/1 v/v).

Interaction of acrithion 2 with $Hg(ClO_4)_2$. Isolation of *N*-methylacridone (1): Acrithion **2** (112 mg , 0.5 mmol) was stirred for about 15 min with $Hg(ClO_4)_2$ (563 mg, 2.5 mmol) at room temperature in DMSO–H_2O (7:3 v/v) buffered at pH 7.4. During this time, the deep yellow color of the solution turned to colorless. The reaction mixture was diluted with water and a nearly colorless solid precipitated. The TLC comparison with the authentic sample, the main product was found to be *N*-methylacridone (**1**) with slight contamination of the unchanged acrithion **2**. The crude product was filtered and crystallization from methanol afforded *N*-methylacridone (**1**, 70 mg; 62.5%), mp 202 °C. (lit. 198–199 °C) [2]; ^{13}C NMR (DMSO-d_6) 177.5, 142.6, 134.8, 126.8, 121.9, 121.6, 116.5, 34.0 ppm.

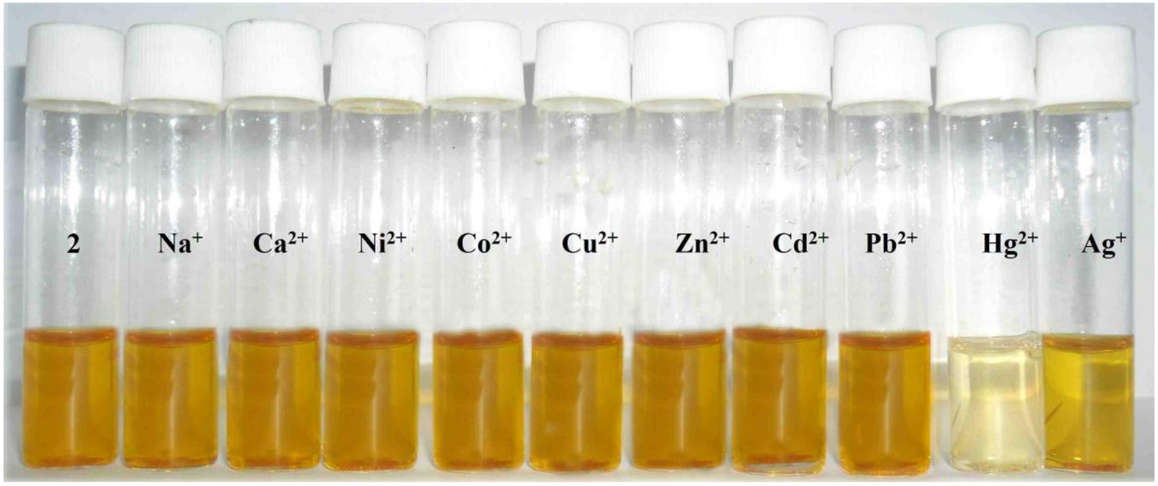

Figure S5: Naked-eye detection of Hg^{+2} by acrithion **2**.

<u>Limit of detection by fluorescence.</u>

The limit of detection was derived using the fluorimetric titration method [3]. The probe, acrithion **2** was employed at a fixed concentration of 1×10^{-8} M and the slit width was adjusted to 5.0 nm/5.0 nm. The signal to noise ratio was evaluated by measuring the emission intensity of blank six times and the standard deviation of blank measurement was determined. As shown in Figure S6, under this conditions, we observed a linear relationship between acrithion **2** and Hg^{2+} (incremental concentration from 2×10^{-9} M to 1.8×10^{-8} M). The detection limit was then calculated using the following equation: Detection limit = σ_{bi}/m, where σ_{bi} is the standard deviation of six blank measurments, *m*

is the slope between intensity vs Hg^{2+} concentration. Accordingly, the limit of detection was calculated to be 1.06×10^{-8} M.

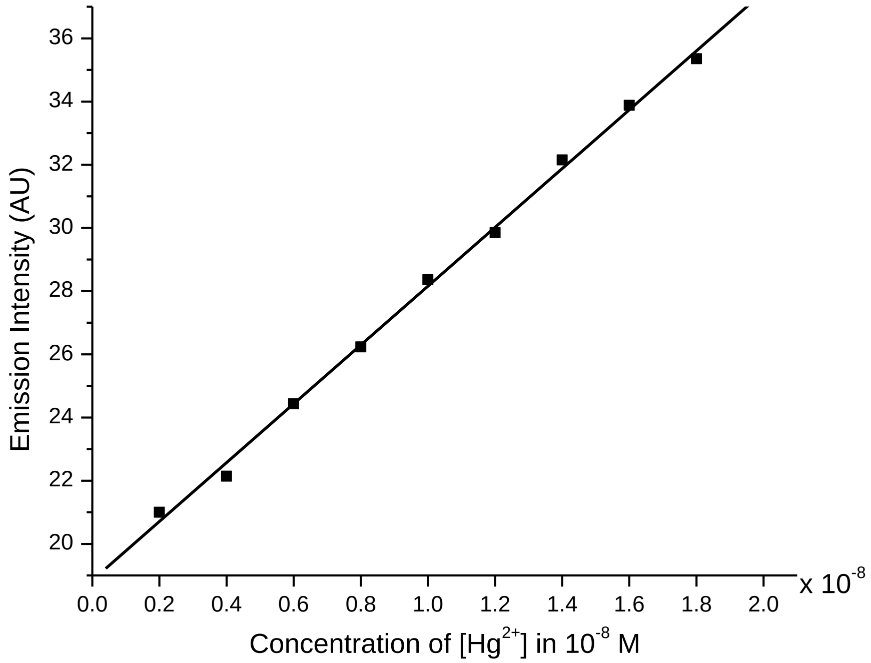

Figure S6: Determination of the detection limit of Hg^{+2} with acrithion **2** by fluorescence spectroscopy.

Table S1: Calculation of detection limit of Hg^{2+} with probe.

σ_{bi}	m	S/N	Detection limit
3.15	8.9×10^{-9}	3	1.06×10^{-8} M

References

1. Mizuyama, K.; Tominga, Y.; Matsuda, Y.; Kobayashi, G. *Chem. Pharm. Bull.* **1979,** *27,* 2879. doi:10.1248/cpb.27.2879
2. Gilman, H.; Spatz, S. M. *J. Org. Chem.*, **1952,** *17,* 860. doi:10.1021/jo01140a012
3. Ono, A.; Togashi, H. *Angew. Chem., Int. Ed.* **2004,** *43, 4300.* doi:10.1002/anie.200454172
